# Supplementary material for: Chiral electrocatalysts eclipse water splitting metrics through spin control
Source: Nat Commun. 2023 Feb 24;14:1067. doi: 10.1038/s41467-023-36703-w (PMC9958132; doi:10.1038/s41467-023-36703-w)
Supplement: Supplementary file 1 — Supplementary Information [file 41467_2023_36703_MOESM1_ESM.pdf]

Supplementary Materials for

Chiral electrocatalysts eclipse water splitting metrics through spin control

Aravind Vadakkayil,[1] Caleb Clever,[1] Karli N. Kunzler,[1] Susheng Tan,[2,3] Brian P. Bloom,[1*] and David H. Waldeck[1,2*]

[1] Chemistry Department, University of Pittsburgh, Pittsburgh, Pennsylvania 15260, United States.

[2] Petersen Institute of Nanoscience and Engineering, University of Pittsburgh, Pittsburgh, Pennsylvania 15260, United States

[3] Department of Electrical and Computer Engineering, University of Pittsburgh, Pittsburgh, Pennsylvania 15260, United States.

*email: bpb8@pitt.edu, dave@pitt.edu

Supplementary Note 1

To ensure that the improvements in reaction overpotential for chiral catalysts, over their achiral equivalents, does not arise from differences in the electrochemical surface area (ECSA), Figure S1 shows representative double layer capacitance measured of undoped L- (filled symbol) and Rac-cobalt oxide (open symbol) for determining the ECSA. Figure S2 shows the ECSA normalized linear sweep voltammograms for undoped (black) and 5% (green), 10% (blue), and 23% iron-doped cobalt oxide (purple). The improvement in chiral catalysts (solid line) is found to persist over that of equivalent achiral catalysts (dashed line) and indicates that the improvement in overpotential in Figure 2 is not associated with differences in the density of catalytic sites.

Supplementary Note 2

To test the stability of the individual catalysts linear sweep voltammograms were measured before and after 2 hours of applying a constant current of 10 mA cm^{-2} under mild stirring conditions. Figure S3 shows the voltammograms before and after the constant current and Table S2 reports the overpotential at 10 mA cm^{-2}.

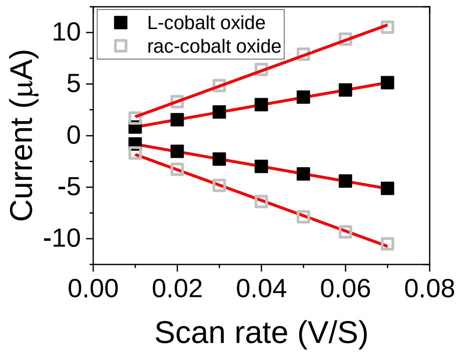

Figure S1. Representative double layer capacitance measurements for undoped L- (filled symbol, black) and rac-cobalt oxide (open symbol, grey) catalysts.

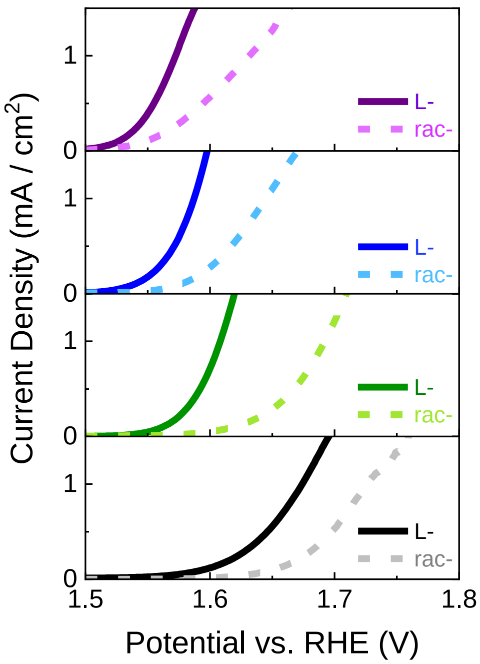

Figure S2. Linear sweep voltammograms of undoped (black) and 5% (green), 10% (blue), and 23% (purple) iron-doped Rac- (light) and L-cobalt oxide (dark) nanoparticle catalysts in Nafion measured in a 1M NaOH electrolyte. The current is normalized to the electrochemical surface area.

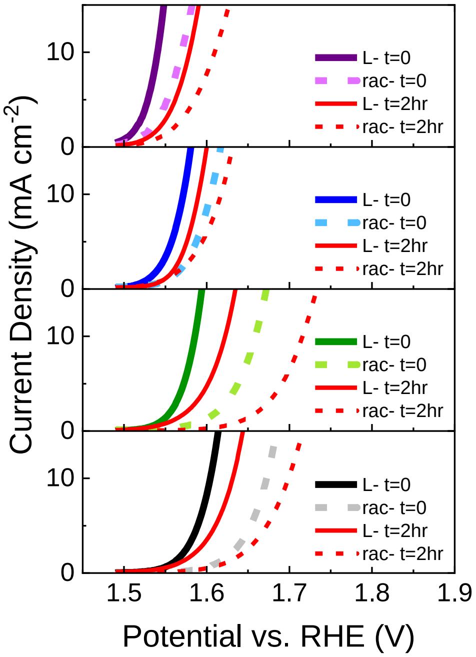

Figure S3. Linear sweep voltammograms of undoped (black) and 5% (green), 10% (blue), and 23% (purple) iron-doped Rac- (light) and L-cobalt oxide (dark) nanoparticle catalysts in Nafion measured in a 1M NaOH electrolyte. Following 2 hours at a current density of 10mA cm^{-2} the linear sweep voltammogram were measured again. The chiral catalyst is indicated by a red, solid line and rac-catalyst by a red dashed line.

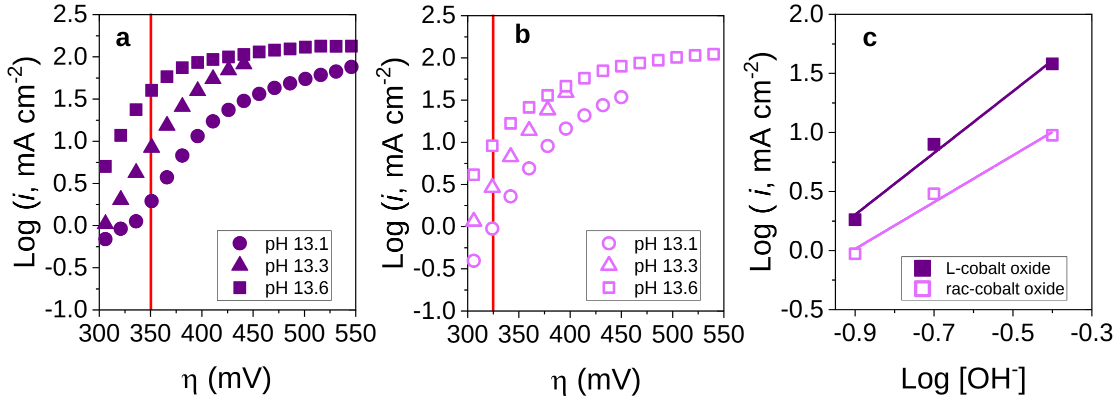

Figure S4. Representative data showing the determination of reaction order. Panel (a) and (b) give pH dependent Tafel plots for 23% L-cobalt oxide and rac-cobalt oxide respectively. The circles, triangles, and squares correspond to pH 13.1, 13.3, and 13.6 respectively. The red line indicates the potential at which the reaction order was determined. The slope for the data plotted in Panel (c) is the reaction order.

Table S1. ICP-OES data for the determination of catalyst stoichiometry.

**Fe Mole fraction (%)**	**Cobalt (ppm)**	**Iron (ppm)**	**Percent Cobalt (%)**	**Percent Iron (%)**
**L - 0**	8.34	0	100	0
**L - 5**	9.72	0.49	95	5

**L - 10**	5.21	0.52	91	9
**L - 25**	3.95	1.09	77	23
**Rac – 0**	7.74	0	100	0
**Rac - 5**	8.87	0.45	95	5
**Rac - 10**	6.46	0.69	90	10
**Rac - 25**	5.13	1.36	78	22

Table S2. summarizes the electrochemical characteristics, at benchmark conditions, for the eight different catalysts synthesized and studied in this work, as well as characteristics for state-of-the-art catalysts previously published in other works.

Table S2. Benchmarking Parameters for Catalyst Materials. The error bars represent the standard deviation generated from measurements of at least three independently prepared electrodes.

**Catalyst**	**ECSA (cm^2)**	**RF**	**η_{geo} t=0hr @ 10mA/cm^2 (mV)**	**η_{geo} t=2hr @ 10mA/cm^2 (mV)**	**Tafel Slope (mV dec^{-1})**	**MA (A g^{-1}) @350mV**	**SA (mA cm^{-2}) @350mV**
**IrO_x***			380 ± 10		48	4.2	0.005 ± 0.003
**IrO_x#**	21 ± 10	105 ± 53	320 ± 40	1050 ± 200			0.4 ± 0.2
**CoO(OH)+**	1.3 ± 0.1				43	~1760	>0.2
**L**	2.1 ± 0.5	30 ± 6	386 ± 11	403 ± 7	59	101 ± 17	0.05 ± 0.02
**5% L**	2.1 ± 0.4	30 ± 5	360 ± 3	397 ± 4	52	232 ± 33	0.26 ± 0.01
**10% L**	1.4 ± 0.5	20 ± 7	339 ± 4	362 ± 1	42	605 ± 84	0.88 ± 0.37
**23% L**	2.9 ± 0.1	42 ± 2	314 ± 3	350 ± 1	34	1730 ± 178	1.18 ± 0.11
**Rac**	3.3 ± 0.2	48 ± 3	435 ± 10	470 ± 1	59	19 ± 12	0.01 ± 0.01
**5% Rac**	2.2 ± 0.4	31 ± 6	408 ± 16	479± 7	62	48 ± 22	0.04 ± 0.01
**10% Rac**	3.0 ± 0.7	43 ± 10	362 ± 11	391 ± 3	52	251 ± 116	0.15 ± 0.06
**23% Rac**	3.5 ± 0.5	49 ± 7	333 ± 5	380 ± 8	42	677 ± 113	0.37 ± 0.04

* Measurements from Ref [1] in 1M NaOH on a GC electrode.
# Measurements from Ref [2] in 0.1M NaOH on a GC electrode.
+ Measurements from Ref [3] in 0.1M KOH on a GC electrode. The mass activity was adjusted to account for the total mass of the catalyst, instead of the metal content alone as in the initial report.

[1] Jung, S., McCrory, C. C. L., Ferrer, I. M., Peters, J. C., & Jaramillo, T. F. Benchmarking nanoparticulate metal oxide electrocatalysts for the alkaline water oxidation reaction. *J. Mater. Chem. A*, **4**, 3068–3076, (2016).
[2] McCrory, C. C. L., Jung, S., Peters, J. C., & Jaramillo, T. F. Benchmarking heterogeneous electrocatalyts for the oxygen evolution reaction. *J. Am. Chem. Soc.* **135**, 16977-16987, (2013).
[3] Hasse, F. T. et al. Size effects and active state formation of cobalt oxide nanoparticles during the oxygen evolution reaction. *Nat. Energy.* **7**, 765-773, (2022)
